# Evaluation of regional ventilation by electric impedance tomography during percutaneous dilatational tracheostomy in neurocritical care: a pilot study

**DOI:** 10.1186/s12883-020-01948-1

**Published:** 2020-10-12

**Authors:** Vera Spatenkova, Eckhard Teschner, Jaroslav Jedlicka

**Affiliations:** 1 Neurocenter, Neurointensive Care Unit, Regional Hospital, Husova 357/10, 46063 Liberec, Czech Republic; 2 Drägerwerk AG & Co. KGaA, Luebeck, Germany

**Keywords:** Electric impedance tomography, Neurocritical care, Percutaneous dilatational tracheostomy, Tracheostomy, Lung imaging

## Abstract

**Background:**

Percutaneous dilatational tracheostomy (PDT) has become a widely performed technique in neurocritical care, which is however known to be accompanied by some risks to the patient. The aim of this pilot study was to assess the derecruitment effects of PDT with the electric impedance tomography (EIT) during the PDT procedure in neurocritical care.

**Methods:**

The prospective observational pilot study investigated 11 adult, intubated, mechanically ventilated patients with acute brain disease. We recorded EIT data to determine regional ventilation delay standard deviation (RVD SD), compliance win (CW) and loss (CL), end-expiratory lung impedance (EELI), with the EIT belt placed at the level of Th 4 before, during and after the PDT, performed in the standard PDT position ensuring hyperextension of the neck.

**Results:**

From 11 patients, we finally analyzed EIT data in 6 patients - EIT data of 5 patients have been excluded due to the insufficient EIT recordings. The mean RVD SD post-PDT decreased to 7.00 ± 1.29% from 7.33 ± 1.89%. The mean post-PDT CW was 27.33 ± 15.81 and PDT CL 6.33 ± 6.55. Only in one patient, where the trachea was open for 170 s, was a massive dorsal collapse (∆EELI − 25%) detected. In other patients, the trachea was open from 15 to 50 s.

**Conclusions:**

This pilot study demonstrated the feasibility of EIT to detect early lung derecruitment occurring due to the PDT procedure. The ability to detect regional changes in ventilation could be helpful in predicting further progression of ventilation impairment and subsequent hypoxemia, to consider optimal ventilation regimes or time-schedule and type of recruitment maneuvres required after the PDT.

## Background

Percutaneous dilatational tracheostomy (PDT) has become a widely performed technique in neurocritical care [1–3]. However, this is a method with some risks, associated with the insertion of a tube to the trachea and derecruitment of the lung. During the opening of the trachea, the closed ventilation system is suddenly disconnected from pressurized mechanical ventilation, and the immediate and subsequent derecruitment of the lung can cause acute hypoxemia and hypercapnia. In neurocritical care, these conditions are especially threatening as they can lead to secondary brain damage and worsened outcome. Therefore, searching for methods increasing safety and reducing risks of PDT is very important in neurocritical care patients. Besides ultrasound [4, 5] or bronchoscopy [6, 7], the electric impedance tomography (EIT) could be considered as another efficient way that is increasing the safety of PDT procedure.

Even though EIT was invented more than 30 years ago, only during the last few years has it started to become widespread as a method assisting safe mechanical ventilation in critical care patients [8–10]. Its major advantage is the radiation-free, non-invasive, bedside and continuous imaging of lungs via real-time EIT-based indices and extended analysis of derived parameters, providing various measures of regional ventilation and its inhomogeneities.

The aim of this pilot study was to detect early signs of lung derecruitment with EIT and to show its potential to monitor adverse effects of PDT procedure on lungs in ventilated patients in neurocritical care. By doing so, EIT can be used to prevent effectively the consequent acute hypoxemia and hypercapnia, which are especially threatening conditions that can lead to the secondary brain damage and worsen outcome and demand immediate bedside reversion.

## Method

### Setting

This pilot study was conducted in the neurointensive care unit (NICU) of the Neurocenter of the Regional Hospital, which has 900 beds and a catchment area of approximately half a million people. The study was performed in Part C of the 18-bed NICU. This part has six ventilated beds for neurological and neurosurgical adult patients. This study was carried out from June 2015 to March 2016. The study protocol was approved by the board of the hospital ethical committee.

### EIT (electrical impedance tomography)

EIT is a non-invasive medical imaging technique visualizing the changes of electrical impedance inside the chest. Respecting the air content changes in alveoli altering the impedance of lung tissue during each breathing cycle, the 2D image of the horizontal section of the chest, representing the map of air content inside lungs can be visualized in real-time mode, along with the derived parameters correlating with the regional ventilation distribution (impedance variations ΔZ correlating well with tidal volumes) and electrical correlates of regional lung compliance (pixel compliance = ΔZ/driving pressure) and its changes (CW – compliance win, CL – compliance loss: representing the gain or loss of pixel compliance [9] between the two readouts). The EIT device used to record our patients was Pulmo-Vista 500 (Dräger, Germany), equipped with the chest belt containing 16 recording electrodes + 1 reference electrode, reaching refresh rates of resulting impedance map 30 Hz. Proper real-time processing and also post-processing of the EIT signal requires fairly continuous recording of impedance with electrodes placed at designated positions of stable electrical connection and its quality can easily be affected by the positioning of the patient as it is the case during performing the PDT procedure. Thus we only analyzed the EIT data that reached sufficient quality level, allowing us to see the dynamical ventilation map evolving reliably in time and herewith to analyze properly the possible derecruitment caused by the PDT procedure.

**Fig. 1:**
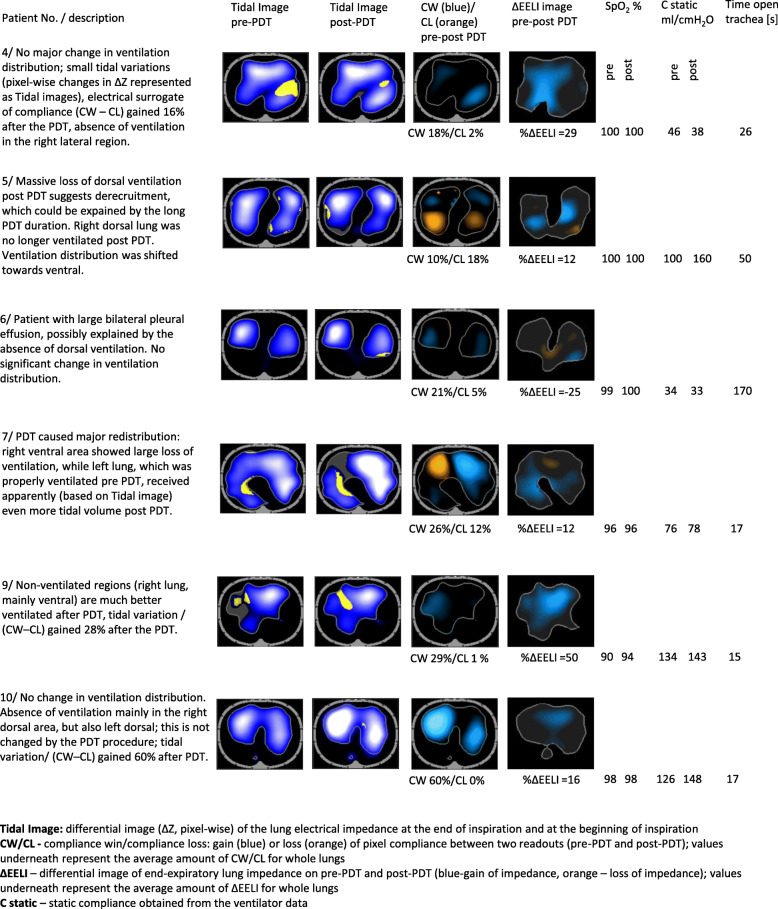
EIT imaging pre and post percutaneous dilatational tracheostomy

Compliance win (CW) and compliance loss (CL), calculated and shown either pixel-wise (Fig. 1) or as an average over all pixels (listed in Table 4), are parameters reflecting how much of the electrical impedance normalized over driving pressure was gained (removed) at given lung region (or EIT map pixel) between two different time instances, and inform us about the electrical surrogate of regional lung compliance as described in details in [9].

RVD: This parameter, called regional ventilation delay correlates with atelectatic areas showing a time delay in the distribution of inspired air during the breathing cycle (was available as a postprocessing parameter). RVD is calculated for each pixel and detailed information about its calculation can be found in [11]. In order to show the RVD for lungs as the whole, the RVD SD parameter was calculated as the standard deviation of all RVD pixels evaluated at a given time instant, providing a mathematical approximation of the inhomogeneity of regional delay times in lungs.

EELI: End-expiratory lung impedance (its change notated as ΔEELI) is an electrical impedance of lungs (pixel-wise shown in Fig. 1 and averaged over all pixels listed in Table 4) at the end of expiration and reflects the air volume inside lungs at the end of expiration and its decrease signalizes possible collapse in dependent regions.

### Study population

The prospective observational pilot study investigated 11 adult (10 men and 1 woman, age range from 34 to 87 years with mean age 68.2 ± 16.1 years), intubated, mechanically ventilated patients (Hamilton Medical, G5) with acute brain disease who had been indicated for PDT. Demographic data, endotracheal tube and time spent on mechanical ventilation prior to the PDT can be seen in Table 1. None of patients had lung disease or lung therapy or nicotine intake in their history.

**Table 1:** Demographic characteristics of the study population and parameters associated with percutaneous dilatational tracheostomy

Patient number	Weight kg	IBW kg	Brain diagnoses	ETT size	V days	MAP torr	NA	Trach tube
1	90	61	Stroke	7.5	3	110	0	8.5
2	140	64	Tumour	8.5	5	75	NA	9
3	80	66	Trauma	8.5	6	86	NA	9
4	77	62	Stroke	8.5	6	110	0	9
5	70	68	Stroke	8.5	5	83	0	9
6	75	60	Trauma	8.5	7	65	0	8.5
7	61	71	Stroke	8.5	7	70	NA	8.5
8	100	76	Stroke	9	9	96	0	9
9	100	64	Stroke	9	7	73	0	8.5
10	90	66	Stroke	9	6	98	0	8
11	75	75	Stroke	9	13	93	0	7.5

*IBW* Ideal body weight, *ETT* Endotracheal tube, *V* Ventilation, *MAP* Mean arterial pressure, *NA* Noradrenaline, *Trach tube* Tracheal tube

### Study design

Percutaneous dilational tracheostomy (PDT) is a standard routine procedure performed at the bedside in the NICU, using Portex® ULTRAperc® PDT kits under analgosedation (sufentanil, midazolam) and relaxation (atracurium), mechanical ventilation, and the pre- and post-procedural examination of the lungs was carried out by X-ray. PDT is performed in the standard position with the spine underlaid from the cervical area downwards. For the purposes of this study, PDT was only conducted by skilled, experienced neurointensivists.

Each patient was connected to the EIT device Pulmo-Vista 500 (Dräger, Germany) approximately 1 h before the PDT was started, during the PDT procedure and a minimum of 4 to 12 h after the PDT. A rubber belt containing EIT electrodes was attached to the patient in the size recommended by the manufacturer at the level of Th 4 and it was wrapped in gauze spread with ECG gel and connected to the machine. We kept the recording of the whole time interval of this EIT examination. We measured EIT in order to detect conditions of lungs before the PDT procedure, to see possible periprocedural complications defined as pneumothorax, pneumomediastinum, atelectasis, time of disconnection of the tube, lung derecruitment, regional ventilation delay standard deviation (RVD SD), compliance win (CW) and loss (CL), end-expiratory lung impedance (EELI), ∆EELI and the consecutive effect of these lung changes on oxygenation and CO_2_ levels. We recorded the size of tracheostomy tube and parameters of mechanical ventilation, oxygenation and end-tidal carbon dioxide concentration (EtCO_2_) from 1 h before PDT (Time T-1), during PDT (Time T1) and after PDT (hourly intervals T2-T8).

## Results

From all 11 patients, 2 had minor bleeding at the surgical site during the procedure. In one case a two-stitch suture was performed, while in the other coagulation was sufficient. PDT was performed on the remaining patients without complications. Three patients received vasopressor noradrenalin during PDT. Demographic data, endotracheal tube and time spent on mechanical ventilation prior to the PDT can be seen in Table 1. Parameters related to mechanical ventilation, oxygenation, EtCO_2_ and others, obtained at various consecutive times and averaged over all patients are shown in Table 2. X-rays before and after PDT, describing radiological findings in individual patients that can be correlated with EIT images (Fig. 1), are shown in Table 3.

**Table 2:** Parameters in eleven patients in percutaneous dilatational tracheostomy

Time	Modes	MV l/min	Ppeak cm H_2_O	VT ml	PEEP cm H_2_O	C static ml/cmH_2_O	FiO_2_%	SpO_2_%	EtCO_2_ torr
T − 1	ASV	10.25 ± 2.62	16.27 ± 3.19	546.91 ± 90.67	5.09 ± 0.29	80.82 ± 32.56	0.41 ± 0.04	97.45 ± 3.00	36.95 ± 4.46
T 1	DuoPAP	10.25 ± 2.20	15.91 ± 4.14	564.45 ± 79.29	5.09 ± 0.29	78.36 ± 41.83	1.00 ± 0.00	98.18 ± 1.95	36.48 ± 5.44
T 2	ASV	10.24 ± 2.33	15.55 ± 3.77	535.45 ± 150.00	5.36 ± 0.88	80.91 ± 37.05	0.45 ± 0.12	97.27 ± 2.34	36.89 ± 4.68
T 3	ASV	10.07 ± 2.29	15.18 ± 4.37	571.82 ± 141.77	5.36 ± 0.88	102.18 ± 49.28	0.44 ± 0.10	98.18 ± 1.59	36.55 ± 3.87
T 4	ASV	9.92 ± 2.03	15.27 ± 3.98	568.45 ± 95.56	5.36 ± 0.88	92.64 ± 35.34	0.42 ± 0.07	98.00 ± 1.95	35.32 ± 3.52
T 5	ASV	13.96 ± 2.62	15.18 ± 4.02	555.64 ± 125.64	5.27 ± 0.62	94.64 ± 31.83	0.42 ± 0.06	98.00 ± 2.17	35.05 ± 2.92
T 6	ASV	10.59 ± 2.33	15.09 ± 4.06	600.18 ± 125.39	5.27 ± 0.62	92.00 ± 31.46	0.42 ± 0.06	97.45 ± 2.57	34.57 ± 4.01
T 7	ASV	10.05 ± 1.95	15.00 ± 3.93	558.00 ± 118.00	5.27 ± 0.62	83.00 ± 32.32	0.42 ± 0.06	98.18 ± 2.04	33.95 ± 3,63
T 8	ASV	10.56 ± 1.70	15.64 ± 3.47	555.18 ± 106.69	5.27 ± 0.62	85.91 ± 33.41	0.40 ± 0.03	97.73 ± 2.05	33.95 ± 4.83

T-1 - one hour before percutaneous dilatational tracheostomy (PDT), T1 during PDT, T2-8 hourly intervals, *Mean ±* Standard deviation, *ASV* Adaptive support ventilation, *DuoPAP* Duo Positive Airway Pressure, *MV* Minute ventilation, *Ppeak* Peak pressure, *VT* Tidal volume, *C* Compliance, *FiO*_*2*_ Fraction of inspired oxygen, *EtCO*_*2*_ End-tidal carbon dioxide concentration, *SpO*_*2*_ Pulse oximetry, *EIT* Electric impedance tomography

**Table 3:** Comparison of X-rays pre- and post-percutaneous dilatational tracheostomy

Patient number	Pre PDT	Post PDT
1	Right basal area: progression of condensation.	Right basal area: bronchopneumonia with higher proportion of basal pleural effusion and hypoventilation.
2	No negative findings.	No negative findings.
3	Right base upper lobe: new inhomogeneous shading. Left caudal lung: pleural effusion and hypoventilation.	Right middle area: minimal regression of inhomogeneous shading. Left middle and lower area: slight regression of lower transparency with spontaneous pleural effusion.
4	No negative findings.	No negative findings.
5	Right lower area: minor progression of inflammation.	Right lower area: slight regression of inflammatory changes.
6	Right basal right area: pleural effusion.	Right middle and lower, left basal area: pleural effusion.
7	Right middle area: inflammation.	Right of upper lobe and left paracardical basal: bronchopneumonia.
8	Right middle area: small residual shadow.	Bilateral, bigger on right: pleural effusion.
9	Bilateral lower area: plate-like atelectases.	Left lower area: plate-like atelectases.
10	Basal right area: lower transparency.	Right basal: partial regression of lower transparency.
11	Bilateral basal area: spilled pleural effusion.	Bilateral, bigger on right, basal area: spilled pleural effusion.

From the 11 patients, 5 were excluded due to the insufficient recordings of their EIT results:

as proper analysis of EIT data requires fairly continuous recording of impedance with 16 electrodes, placed at designated positions, of a good and stable connection and conductance with the skin, all recordings substantially affected by the positioning of the patient during securing the standard PDT position were discarded to assure proper evaluation of the EIT data. Varying alignment of electrodes due to the belt shifted downwardly (approaching undesirable diaphragmatic position) happened in three patients, rotation of the belt around the chest in one patient, and problematic interference of EIT with PDT procedure (occurred in one patient) were the reasons why EIT data were not included and analyzed. In Table 4 we present the EIT parameters of the remaining 6 patients. Mean RVD SD post-PDT decreased to 7.00 ± 1.29% from 7.33 ± 1.89%. Mean post-PDT CW was 27.33 ± 15.81 and mean PDT CL 6.33 ± 6.55. Only in one patient, where the trachea was open for 170 s, was a massive dorsal collapse (∆EELI − 25%) detected.

**Table 4:** Data of electric impedance tomography

Patient number	RVD SD %		Pre-post PDT CW/CL		ΔEELI % of pre PDT	Time open trachea s	C static ml/cmH_2_O		SpO_2_%	
	Pre PDT	Post PDT					Pre PDT	Post PDT	Pre PDT	Post PDT
4	9	6	18	2	29	26	46	38	100	100
5	10	7	10	18	12	50	100	160	100	100
6	7	7	21	5	−25	170	34	33	99	100
7	7	8	26	12	12	17	76	78	96	96
9	7	9	29	1	50	15	134	115	90	94
10	4	5	60	0	16	17	126	120	98	98

*PDT* Percutaneous dilatational tracheostomy, *RVD SD* Regional ventilation delay standard deviation, *CW* Compliance win, *CL* Compliance loss, *ΔEELI* End-expiratory lung impedance change, *s* Second, *C* Compliance, *SpO*_*2*_ Pulse oximetry

Table 4 summarises some findings which are depicted pixel-wise as 2D images in Fig. 1. E.g. the scalar values of CW/CL represent the average amount of CW/CL of whole lungs obtained by pixel-wise averaging of CW/CL images (matrices) shown in Fig. 1 and demonstrate the so-called pixel compliance change, which is considered as the EIT correlate of lung compliance change. We can see, that after the PDT the CW prevails over the CL, indicating the lung ventilation improved in most of patients. The pre-post PDT RVD SD shown in Table 4 and in Fig. 1 reflects the homogeneity of ventilated areas (taking into account the distribution of collapsed regions) and its value improved after the PDT in most cases. An interesting aspect of EIT data inspection are the pre- and post- PDT tidal images (see Fig. 1) representing the pixel-wise regional lung ventilation via tidal variation of ΔZ. Similarly, changes in EELI, shown as ΔEELI images in Fig. 1, represent the changes of volume of air inside lungs at the end of expiration and are capable of revealing suspected regions of collapse (detected partially in patient 6, whereas the rest of patients showed an overall increase in end-expiratory air volume). It is worth mention that changes in tidal images and ΔEELI are not always correlated. Both the increase of ventilation and the increase of lung volume may indicate improved lung conditions (such as compliance), but the effects are often occurring in different lung regions.

Overall, the PDT resulted in an increase in lung end-expiratory volume and larger tidal volumes. However, in two patients with longer PDT times, the lung conditions got worse in two different ways: while patient 6 showed a significant drop in lung end-expiratory volume, patient 5 presented a loss of dorsal tidal volumes, leading to dorsal lung collapse and more inhomogeneous ventilation distribution.

## Discussion

Nowadays, percutaneous dilatational tracheostomy (PDT) is commonly used in neurocritical care [1–3]. However, while effective, this method has several potential pitfalls and can lead to serious complications. Therefore, various ways are being sought to make this procedure safer. Bronchoscopy and ultrasound [4–7] are two bedside methods that improve safety during the insertion of the tube to the trachea, but a methodology for the continuous imaging of derecruitment of the lungs as a potential risk of hypoxemia is missing. We see a solution to this absence at the bedside, a continuous, non-invasive and radiation-free EIT, which has been becoming increasingly widespread in recent years in critical care [12].

In neurocritical care, special attention must be paid to the elimination of risks potentially leading to hypoxemia, as this could cause secondary brain damage. For this reason, we conducted this first pilot study to try out EIT imaging to improve PDT safety. The expected advantage of this method was the real-time non-invasive and continuous imaging of lung function during these procedures to determine ventilation lung inhomogeneity and loss of lung volume, which have been reported to be indicators of lung derecruitment [11, 13].

Our data suggest that EIT can allow timely detection of immediate changes in lung function for early intervention. We think that such immediate, bedside, and timely detection, enabled by the EIT monitoring, can be crucial because it allows an early intervention as well as an early, timely and immediate evaluation of the responses to the intervention. Although pulse-oximetry allows very fast, simple and continuous monitoring for the detection of hypoxemia, EIT can provide additional information for decision making regarding the causes of the hypoxemia that may result in different interventions. E.g., based on the localization of the collapse (dorsal vs. ventral, right side vs. left side), the proper positioning of the patient can be imposed - favoring (due to the effect of gravity on lungs) or optimizing the ventilation of atelectatic region without the need of more invasive ventilation intervention. Moreover, EIT assisted optional recruitment procedure performed right after the PDT can help optimizing the PEEP value, respecting the localization and extend of ventilation impairment.

In this study, as our results show, the EIT imaging could only be assessed in 6 patients. We see the reason for this in that we were just starting to use this method, and the placing of the belts around the chest is not entirely simple in the situation when we underlay the spine from the cervical area downwards with a rolled sheet, the belts can shift and the electrodes in the transition zone between the rolled sheet and the mattress may lose contact. Since the proper analysis of EIT data requires a fairly continuous recording of impedance with 16 electrodes of a good and stable electrical connection, all recordings substantially affected by the positioning of the patient during the PDT were discarded to assure proper evaluation of the EIT data. So, the EIT of three patients was not included due to the unwanted diaphragmatic position of the belt, in one patient was the cause the rotation of the belt and in one patient the PDT procedure interfered substantially with EIT recordings. However, this is the standard patient position during PDT in our neurocritical care, so we did not change this underlaying of patients and at the same time we did not want to interrupt the procedure (using a gel pad or pillow slightly broader than the rolled sheet could partially eliminate this issue in the future). Nevertheless, meaningful data from all patients who consecutively underwent the PDT procedure were analyzed and discussed in this study, mainly to address and communicate described difficulties with the belt position and to discuss the appropriate care required to ensure a good signal quality in order to obtain reliable EIT imaging.

Furthermore, in this pilot study, we wanted to eliminate physician error, and so PDT was performed by skilled physicians using standard methods. This can be seen in our EIT results, except for the two cases when there were short bleedings in the PDT wounds. Otherwise, all PDTs were performed without any complications. We also see that the time the trachea was kept open was mostly very short, the longest was 170 s. This duration is reflected in the EIT images, where we detected a massive dorsal collapse (∆EELI − 25% in patient number 6). In patient number 5, we see that some lung tidal volume was lost, but compliance was not impaired significantly. The remaining four patients had good EIT images, with increases in lung end-expiratory and tidal volumes.

Our EIT imaging results show the benefits of this method for an individualized approach in detecting derecruitment (we only saw derecruitment in one case, but this patient’s PDT lasted longer). Early detection of derecruitment would be beneficial for increasing the safety of PDT procedures – seeing rapid and massive derecruitment during the PDT provides an important information about the tendency of lungs to collapse. Such information regarding the scale, localization and dynamic of atelectasis formation detected by EIT during the PDT – conceived as a stress test of lungs in ventilated patients - can be very valuable in a consecutive adjustment of proper ventilation settings after the PDT, taking into account tendency of lungs to collapse and regions prone to atelectasis. The real-time 2D maps of electrical impedance and derived parameters (CW, CL, RVD, ΔEELI) containing pixel-wise information about the localization and scale of the ventilation disturbances, deployed with appropriate interpretation, provide valuable correlate of spatial or timeous ventilation homogeneity and could be of substantial value when adjusting PEEP values and setting ventilation schemes reducing regions of atelectasis and optimizing the ventilation.

A further advantage of proposed EIT monitoring is that one X-ray shot, the post PDT radiation exposure, can be avoided because EIT monitoring provides real-time information on changes in ventilation distribution and lung volumes, timely and at the bedside. However, we present data from a pilot study, and therefore further research is needed to determine the relationship between an open trachea and derecruitment and to confirm the potential usefulness of this technology for improving PDT safety in neurocritical care. We believe that further EIT research defining additional, advanced real-time parameters with promising potential in EIT assisted ventilation intervention will further improve outlined solutions dealing with the adverse effects of derecruitment during the PDT.

## Conclusion

This pilot study shows that EIT imaging is a potentially useful method for improving safety during the PDT procedures by allowing a bedside, early and timely detection of immediate changes in lung function for early intervention.

## Data Availability

The datasets obtained during this study are available from the corresponding author on reasonable request.

## Abbreviations

ASV: Adaptive support ventilation
DuoPAP: Duo Positive Airway Pressure
C: Compliance
CL: Compliance loss
CW: Compliance win
ΔEELI: Delta End-expiratory lung impedance
EIT: Electric impedance tomography
ETT: Endotracheal tube
F: Female
EtCO_2_: End-tidal carbon dioxide
FiO_2_: Fraction of inspired oxygen
IBW: Ideal body weight
M: Male
MAP: Mean arterial pressure
SD: Standard deviation
MV: Minute ventilation
NA: Noradrenaline
PDT: Percutaneous dilatational tracheostomy
Ppeak: Peak pressure
RVD SD: Regional ventilation delay standard deviation
s: Second
SpO_2_: Pulse oxymetry
T-1: One hour before PDT
T1: During PDT
T2–8: Hourly intervals
Trach tube: Tracheal tube
V: Ventilation
VT: Tidal volume
